# Agar Biopolymer as a Sustainable Alternative Binder to Enhance the Strength of Low-Plasticity Soil

**DOI:** 10.3390/polym17091253

**Published:** 2025-05-05

**Authors:** Mary Ann Adajar, Jomari Tan, Adriann Adriano, Sophia Bianca De Vera, John Vincent Manabat, Harumi Navarro

**Affiliations:** Department of Civil Engineering, De La Salle University, Manila 1004, Philippines; jomari.tan@dlsu.edu.ph (J.T.); adi_adriano@dlsu.edu.ph (A.A.); sophia_bianca_devera@dlsu.edu.ph (S.B.D.V.); john_vincent_manabat@dlsu.edu.ph (J.V.M.); harumi_navarro@dlsu.edu.ph (H.N.)

**Keywords:** agar biopolymer, low-plasticity soil, cement binder, unconfined compression strength, masking effect, soil stabilization

## Abstract

Low-plasticity silts (ML) found in Metro Manila, Philippines, characterized by low strength, stiffness, and bearing capacity, often require stabilization. Traditional methods using cement are associated with significant carbon emissions, causing environmental concerns. Sustainable materials such as agar biopolymers can be an alternative to cement to improve the strength of fine-grained soils. A comparative study was conducted on ML samples treated with agar and cement at different concentrations (1%, 3%, 5%, and 7%) and subjected to varying curing periods (7, 21, 28, and 35 days) under air-dried conditions using Unconfined Compressive Strength (UCS) tests. Agar-treated samples generally exhibited higher UCS values than cement-treated samples across the tested concentrations and curing periods. Samples with 3% and 5% agar were significantly stronger than their cement-treated counterparts. The strength of agar-treated soils peaked at a 5% concentration and subsequently decreased at 7% agar, possibly due to a masking effect. SEM-EDS analysis revealed that a 5% agar concentration achieved a balanced microstructure with effective particle bonding, while higher concentrations led to diminished strength due to reduced mechanical interlocking from excessive biopolymer coverage. Subsequent statistical analysis also indicated significant improvement using agar versus cement-treated and untreated soils, especially at 5% agar. A predictive polynomial regression model demonstrated the influence of curing days and agar concentration on UCS, attaining R^2^ = 0.94 vs. experimental values. Using agar biopolymers presents a promising and potentially more sustainable approach to soil, highlighting the potential of utilizing a locally abundant resource for geotechnical engineering applications.

## 1. Introduction

Soil stabilization is an essential measure in geotechnical engineering aimed at enhancing the strength, durability, and load-bearing capacity of underlying strata while reducing their plasticity and shrink–swell behavior [1]. Stabilization techniques ensure the stability and longevity of structures built on various soil types, especially in areas with weaker fine-grained soils, such as low-plasticity clays. Fine-grained soils, such as silt and clay, are a critical component in the design of any structure due to their inherent properties and response to external loading. Characterized by their minuscule particulate matter, a significant degree of plasticity, and increased water retention capacity, silts and clays exhibit poor shear strength and high compressibility levels. These attributes pose challenges in constructing buildings and fixtures, possibly leading to excessive settlement and instability of structures [2]. This concern becomes even more critical when structures in developed regions expose a dense urban population to potential risks of damage and casualty, thus necessitating various stabilization techniques to improve soil performance. Rapid urbanization in developing regions increases exposure to weak strata in previously undeveloped lands, as existing structures have occupied prime locations [3]. In the metropolitan region of Manila in the Philippines, three major categories of soils exist—clay loam, loam, and sandy loam. Additionally, low-plasticity silt and clay are considered one of Metro Manila’s most prevalent soil types [4]. The saturation of these soils in areas with shallow water tables can exacerbate issues with soil compressibility and settlement [5].

Addressing these issues may involve different soil stabilization approaches. Problematic soil can be replaced with borrowed material sourced from other locations [6]. Fiber reinforcements such as geogrids and geotextiles may be incorporated in retaining walls, foundations, slopes, and embankments [7]. Mechanical approaches often use a mixture of various aggregates to achieve a specified design requirement [8]. Chemical methods also feature the addition of cement, lime, and other industrial byproducts as typical materials for stabilization [9]. Newer methods also utilize polymers of organic and inorganic origin leverage the advantage of molecular chain structures to create durable and economic means of enhancing soil strength [10]. However, chemical and synthetic methods have significant environmental drawbacks associated with high anthropogenic carbon emissions from cement and lime production [11] and increased pH levels in the soil and adjacent groundwater basins [12]. Other approaches such as soil replacement, mechanical stabilization and the use of reinforcing materials are effective yet may not be practical in terms of cost and logistics in certain situations. Sustainable alternatives may be explored from natural materials to promote effective geotechnical engineering solutions while ensuring low environmental impact.

An emerging approach in soil stabilization incorporates biopolymer additives extracted from organic materials such as plants, animals, and microorganisms into weak cohesive soils. Biopolymers possess a soluble chain-like molecular structure whose mechanism involves physical and chemical modifications to the soil structure, improving stability. These polymers enable bio-capsulation and bio-coating due to the electrostatic bonding of water and mineral ions in clayey soils [13], forming a gel-like matrix that binds soil particles together, thereby increasing soil cohesion and reducing susceptibility to erosion. Furthermore, certain biopolymers contribute to bio-cementation by facilitating microbial-induced carbonate precipitation, mimicking natural soil cementation processes, and further reinforcing soil stability [12,14]. The use of biopolymers in soil stabilization has found applications in various engineering and environmental contexts, including road and pavement construction, erosion control, agricultural soil improvement, and land reclamation [15,16]. Their biodegradability and non-toxic nature make them an environmentally sustainable choice compared to conventional chemical stabilizers [17]. Notable examples of biopolymers in geotechnical applications include agar gum, lignin, xanthan gum, dextran, chitosan, guar gum, alginate, casein, starch, and beta-glucan [12,14,18,19,20]. Among these, agar biopolymers have gained considerable interest due to their abundant availability, non-toxic nature, and ability to enhance soil strength [21].

Agar biopolymer is a versatile natural polysaccharide sourced from red algae. It is a thickening agent for its gel-like properties across various applications such as food, medicine, cosmetics, and agriculture [20,22]. Red algae originate from seaweed under the *Rhodophyceae* class, including *Gracilaria*, *Gelidium*, *Gracilariopsis*, *Pterocladia*, and *Gelidiella* [20]. However, the minuscule size and amount of biomass obtained from *Gelidium* and *Pterocladia* reduce the economic feasibility of agar extraction from species under these genera [23]. Hence, agar production is primarily sourced from either *Graciliaria* or *Gelidium*. In the Philippines, *Gracilaria* is one of the prime sources of agar, with at least 18 documented species found within the country [24]. Locally known as *guraman, guraman-dagat, or gara-garao*, *Gracilaria firma,* and *Gracilaria heteroclada* are known for their abundance and quality among all locally cultivated species [25].

The crystalline structure of agar exhibits appreciable strength and rigidity, befitting its potential to become an alternative for soil stabilization. The substance comprises two main components: agarose and agaropectin. The former is responsible for the gelling between the soil particles, while the latter allows for the material’s viscosity [26]. After curing, the agarose content also lets the gel coat the soil particles in a thick film. This forms a complex three-dimensional gel network within the soil matrix, which increases mechanical strength, cohesion, and resistance against deformation while reducing permeability. In addition to this, it also becomes difficult for bacteria to digest the substance. This slows down the biodegradation of the material, allowing it to maintain the soil’s stability for relatively longer periods [27].

Experimental studies in the existing literature have demonstrated that soil samples treated with agar biopolymer can achieve higher compressive strength values than those stabilized with conventional cement-based binders at optimal concentrations. Chang et al. [20] treated clayey soils with 1% agar gum concentration, reaching a maximum increase in unconfined compressive strength by 750 kPa after submergence for varying curing periods. Smitha and Sachan [26] also reported increased shear strengths after short curing periods, which were attributed to a quick gelation process and the absence of reaction during soil cementation. Kantesaria et al. [28] determined that the shear strength doubled and swell pressure decreased by 85% upon adding 3% agar in high-plasticity soils cured for 7 days.

While biopolymers have shown great potential to be a sustainable alternative to cement binders in soil stabilization, they have not been used widely in geotechnical applications due to a lack of characterization of their engineering behavior and a limited set of methodologies that incorporate them in the geotechnical design [14]. Promoting alternative solutions to problematic soils requires a clear comparative analysis highlighting the advantages of biopolymers versus traditional options. In this study, the impact of the chemical bonding of agar to low-plasticity soil particles is quantified by determining the unconfined compressive strength of agar biopolymer-treated soils versus untreated and cement-treated soils. Utilizing agar extracted from local seaweed as a primary additive in soils may ensure better soil capacity to support typical low-rise structures.

## 2. Materials and Methods

### 2.1. Untreated Soil

The soil assessed in this study was sourced from a construction project in Malate, a district in Manila, Philippines. This area was identified to possess a soil profile that features underlying layers of sand, silt, and organic fines [4]. This classification was confirmed through Atterberg limit tests and grain-size analysis following ASTM D4318 [29] and ASTM D7928 [30]. Laboratory tests were performed to determine the index properties of untreated soil specimens and confirm the soil classification. Samples were ensured to be classified as low plasticity before treatment and curing for strength tests. ASTM D2487 [31] stipulated that particle-size characteristics, liquid limit, and the plasticity index influence soil classification. Other parameters, such as specific gravity, maximum dry unit weight, and optimum moisture content, also indicate key information on the effect of any admixture in soil stabilization. Table 1 presents the physical properties of the untreated soil utilized in this study.

### 2.2. Cement Binder

Portland cement Type I was used and mixed with soil samples at 1%, 3%, 5%, and 7% proportion by dry weight at 7, 21, 28, and 35 curing days. The cement binder was included as a reference in comparing the strength performance of soil treated with agar biopolymer.

### 2.3. Agar Biopolymer

The agar biopolymer used in this study was bacteriological-grade agar powder derived from red seaweeds (including *Gracilaria* seaweeds). The agar powder has a gelling strength greater than 800 g/cm^3^ [34], which resembles the gel strength of *Gracilaria heteroclada* at 892 g/cm^2^. Agar concentrations were 1%, 3%, 5%, and 7% by dry weight at 7, 21, 28, and 35 curing days.

### 2.4. Experimental Setup

**Figure 1:**
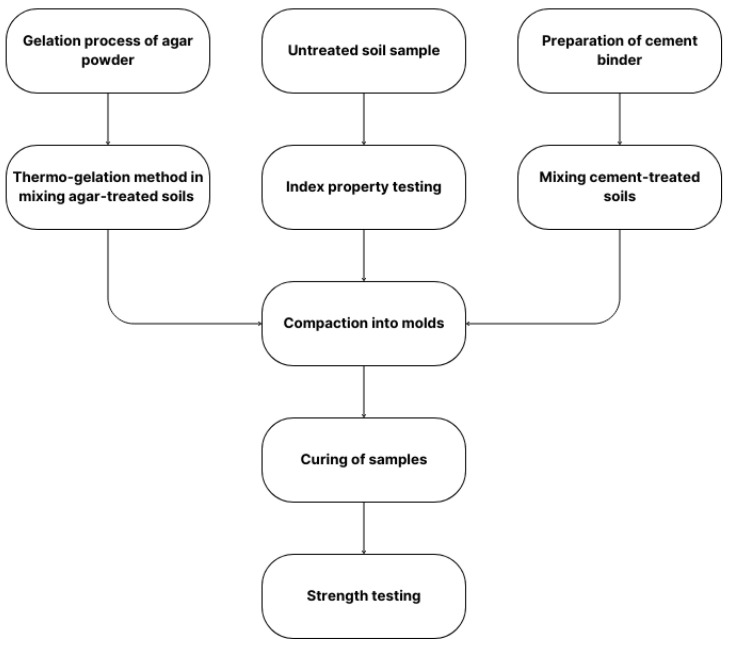
Methodological sequence of the study.

The study aimed to determine the effect of agar biopolymer on the unconfined compressive strength of low-plasticity silt samples. Varying concentrations of agar biopolymer and cement binders were used to compare the performance of traditional stabilizers versus sustainable materials. Four different curing periods (7, 21, 28, and 35 days) were applied to all specimens at average room temperature (24 °C to 26 °C) before the unconfined compression tests. At least three specimens were made for each concentration proportion and each curing period. The sequence of tests performed in this study is shown in Figure 1.

### 2.5. Sample Preparation

#### 2.5.1. Agar Biopolymer-Treated Soil

The gelation of agar powder was achieved by dissolving it in hot water and allowing it to form a stable hydrogel. The process started by heating distilled water to 90 °C. The amount of water for sample preparation must yield a 2% agar solution. Once brought to a boil, the required amount of agar powder for the solution was dissolved in the distilled water. The solution was maintained at a temperature of 90 °C.

The mixture was stirred continuously to promote uniform dissolution and to allow some evaporation of excess water. The amount of evaporated water was not quantitatively measured. It was controlled by visual assessment of the viscosity of the solution, ensuring that the mixture remained fluid but viscous enough to prevent slurry formation during soil mixing. The required dry soil was added to the mixture at the same stirring rate. To ensure the necessary moisture content of the sample, the mixture was weighed before compaction. The resulting weight should represent the total weight of the soil-agar mixture at its optimum moisture content. The soil was quickly compacted into a split-type mold with dimensions of 50 mm in diameter and 100 mm in height in five equal layers at 25 blows each by hand tamping. Excess soil was scraped off the top to ensure the ends were leveled. At the end of the sample preparation, the treated samples were air-dried at room temperature for the required curing days.

#### 2.5.2. Cement-Treated Soil

The required amount of oven-dried soil was mixed with water at optimum moisture content and placed in a sealed plastic container to prevent moisture loss. The soil was cured in this condition for 24 h to allow full water absorption within the soil particles.

The cement powder was mixed with water at a water-to-cement (w/c) ratio of 0.80. The cement powder was thoroughly mixed to ensure dissolution. The 24 h soaked soil sample was poured into the cement mixture for further blending until the soil particles exhibited an evenly distributed light color, as the cement had a light grayish hue. After mixing, the treated specimens were compacted into 50 mm by 100 mm split-type molds at five layers at 25 blows for each layer, which was the same process as the soil–agar mixture.

### 2.6. Unconfined Compression Test

ASTM D2166 [35] outlined the laboratory procedure for determining the unconfined compressive strength (UCS), which can indicate the short-term shear strength of cohesive soil specimens. The strength values calculated from the test results were used to compare the performance of untreated, agar-treated, and cement-treated samples. Results from the compression tests were also used to formulate the predictive model for the strength values.

Provisions from ASTM D2166 require cylindrical samples to have a height-to-diameter ratio of 2 before compressive axial loading in the universal testing machine at an axial strain rate of 0.5% to 2% per minute. Digitally logged raw data for load and deformation were used to generate a stress–strain curve where the peak or the load per unit area at 15% axial strain, whichever occurs first, is considered the specimen’s unconfined compression strength (UCS). The main equation used for this test is

(1) $$q_{u}=\frac{P}{A}$$

where

*q_u_* = unconfined compressive strength

*P* = axial load at failure

*A*= corrected area = *A_o_*/(1 − ε)

*A_o_* = initial area of the specimen

ε = axial strain

The unconfined compressive strength of soil was correlated to its undrained shear strength, which is one-half of its value. Understanding the undrained shear strength of soil is critical for geotechnical engineering applications as it provides insight into a soil’s strength behavior under rapid loading, making it essential for ensuring structural stability and safety in engineering projects.

## 3. Results

### 3.1. Modes of Failure

**Figure 2:**
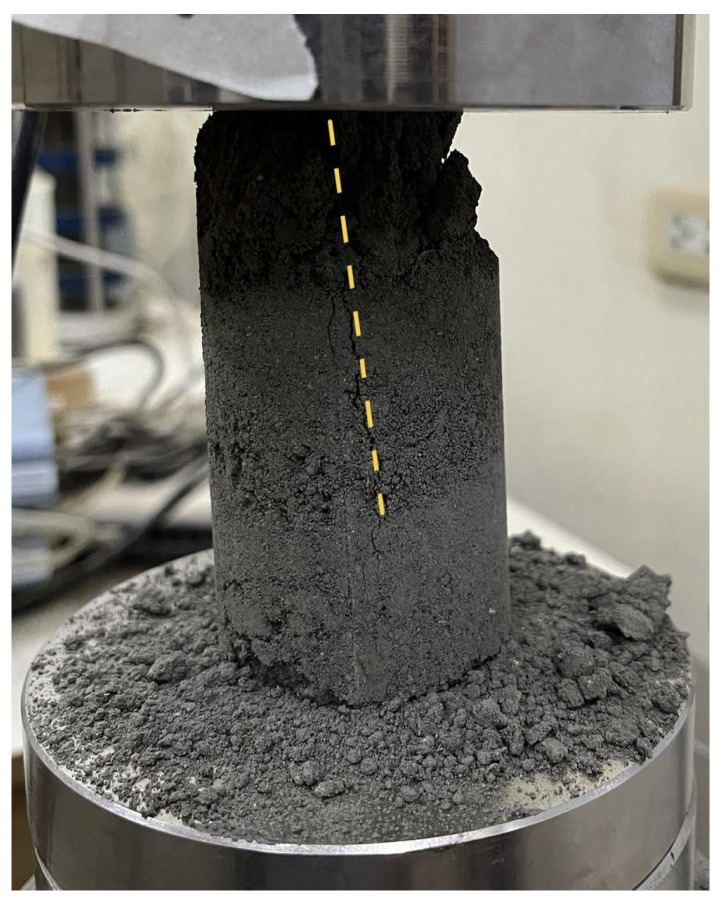
Failure mode observed from an untreated soil sample.

The failure modes of the samples were documented after conducting the uniaxial compressive test. All samples experienced shear failure through single or columnar failure planes and ‘Y’ cone split, which resulted from axial splitting. This refers to the vertical fracturing along the longitudinal axis during axial compression. Under axial compression, the maximum shear stress develops along the failure planes. When shear stress exceeds the material’s shear strength, failure initiates along that plane. The absence of lateral confinement allows radial expansion as the specimen is compressed vertically. This induces tensile hoop stress in the material. When these tensile stresses exceed the specimen’s tensile strength, vertical cracks propagate parallel to the loading direction, causing the specimen to split into columnar fragments [36], indicating weak or low compressive strength values and contributing to the brittle shear mechanism. Cohesive soils with high stiffness and low moisture content often exhibit this failure mode, given that the lack of moisture results in a more flocculated soil structure. Figure 2 shows the mode of failure of an untreated soil sample. A single, inclined shear plane (highlighted by a dashed yellow line) forms across the specimen. This indicates a brittle fracture behavior with little to no plastic deformation. The upper part of the specimen showed material disintegration, as evidenced by the loose particles around the base. The crack propagates abruptly, indicating a sudden failure after experiencing the peak load.

**Figure 3:**
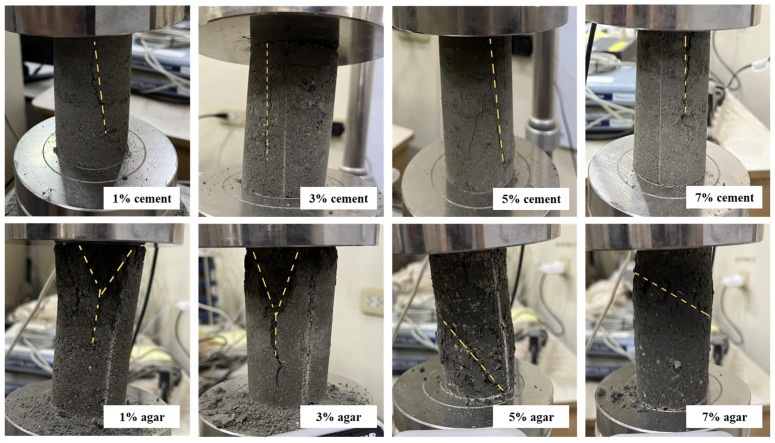
Failure modes in cement-treated and agar-treated soil samples.

Figure 3 illustrates the failure modes for cement-treated and agar-treated soil, respectively. Cement-treated soil with 1% cement content demonstrates a diagonal shear failure with pronounced crumbling. This indicates a brittle failure with wide cracks and visibly disintegrated specimens. Low cement content provides minimal cohesion, which is insufficient for a sample to demonstrate ductile behavior. The sample with 3% cement content fails by diagonal shear but with less fragmentation. Crack is present but less open, transitioning from highly brittle to moderately cohesive.

Adding cement content improved the bonding between soil particles due to the higher cementitious matrix, enhancing integrity. At 5% cement content, shear failure in a columnar plane with finer, less prominent cracks results. The behavior is somewhat more ductile; the specimen holds shape better with visible crack patterns but with less collapse. The cement provides better load transfer and improved cohesion. At 7% cement content, the specimen demonstrated high compressive resistance and ductile failure, showing only minor shear cracking and maintaining structural integrity throughout most of the loading phase. High cement content significantly enhances bonding and strength, improving post-peak behavior and reducing brittleness. The failure mode in cement-treated soil specimens under uniaxial compression can transition from brittle diagonal shear failure at low cement content (1% to 3%) to ductile behavior at higher cement content (5% to 7%). Increasing the cement content strengthens the soil matrix, delays crack initiation, reduces fragmentation, and enhances ductility, resulting in improved structural integrity and performance.

On the other hand, each agar-treated specimen shows a distinct failure pattern indicative of how increasing agar content affects the mechanical behavior and the failure mechanism of the soil mixture. At 1% agar content, axial splitting with a Y-shaped crack occurs, indicating a tensile failure. Tensile splitting is common in weak, low-cohesion soils under uniaxial compression [36]. Soil failing through axial splitting (columnar or ‘Y’ cone) generally has lower strength than those failing through diagonal shear [36]. At low agar concentrations, the binding effect is minimal, and the sample fails primarily due to tensile stresses caused by Poisson’s effect during axial compression. The material has insufficient cohesion and internal friction to resist tensile splitting. 3% agar content led to axial cracking with reduced crack width, indicating some cohesive resistance. Slight biopolymer addition increases ductility, but tensile failure remains dominant [37]. The material begins to exhibit more ductile behavior at 5% agar content. A clear diagonal shear plane develops across the specimen, indicating a diagonal shear failure, which can be observed in improved soils with moderate cohesion [38]. The increase in agar concentration strengthens the soil matrix, allowing it to mobilize shear resistance. The transition from tensile to shear failure indicates that the material can now distribute stress more evenly, and failure occurs along the plane of maximum shear stress. At 7% agar content, the specimen shows a more pronounced diagonal cracking with visible bulging along the sides. This indicates that the sample fails by shear failure with plastic deformation. At higher agar concentrations, the soil exhibits ductile behavior due to the strong interparticle bonding provided by the biopolymer. The sample undergoes more plastic deformation before failure. The failure mode is governed by shear, but the visible bulging suggests enhanced energy absorption and plasticity [39]. As the agar concentration increases, the failure mode transitions from brittle axial splitting to ductile shear failure. This progression reflects enhanced cohesion, energy absorption, and ductility due to the biopolymer’s binding effect. Higher agar content strengthens the soil matrix, enabling it to resist uniaxial compression more effectively and fail through more desirable, energy-dissipating shear mechanisms rather than brittle tensile cracking. This suggests that agar biopolymer is effective in improving the mechanical behavior of soil, especially at concentrations beyond 3%.

### 3.2. Effectiveness of Curing Days

The study investigated the effectiveness of different curing days (7, 21, 28, and 35 days) on the strength properties of untreated, cement-treated, and agar biopolymer-treated low-plasticity clay soils, as determined by the unconfined compressive strength (UCS) test.

**Figure 4:**
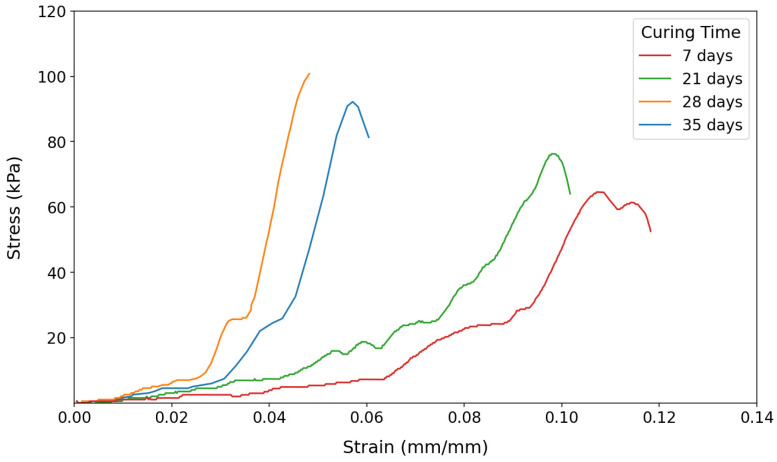
The stress–strain curve for untreated soils.

The results generally indicate that the strengths of all soil samples increased as the curing period lengthened. This is attributed to moisture loss in air-dried samples, increasing effective strength. Figure 4 illustrates this trend for untreated soils, which served as the baseline for succeeding compression tests for treated samples.

**Figure 5:**
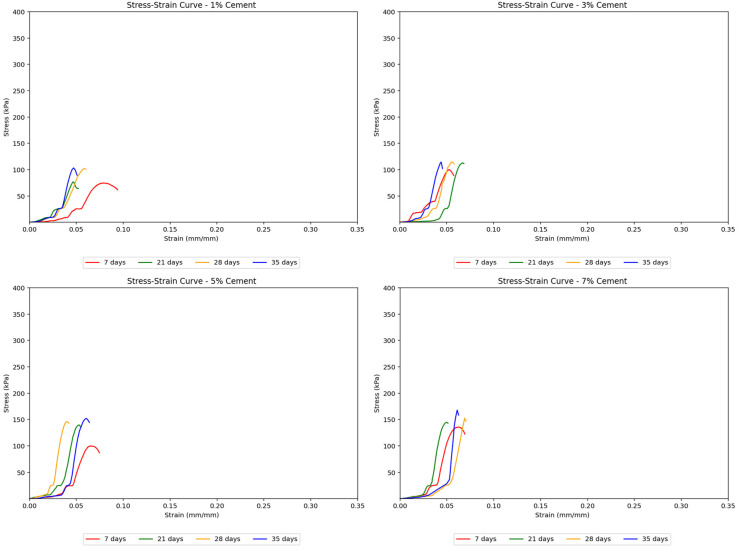
Effect of curing period on the stress–strain curves for cement-treated soil samples.

Cement-treated samples exhibited a more distinct elastoplastic behavior. While a linear stress–strain curve segment was observed in the elastic zone, the curves show a continuous increase in strain at nearly constant stress, thus indicating the yielding of samples. Beyond this threshold, the soil begins to yield and display plastic behavior. The moisture loss in these samples led to steeper stress–strain curves at longer curing periods, suggesting increased stiffness and more brittle behavior, although their peak strengths also increased. Figure 5 shows the effect of cement content on stiffness across different curing days, indicating this trend.

**Figure 6:**
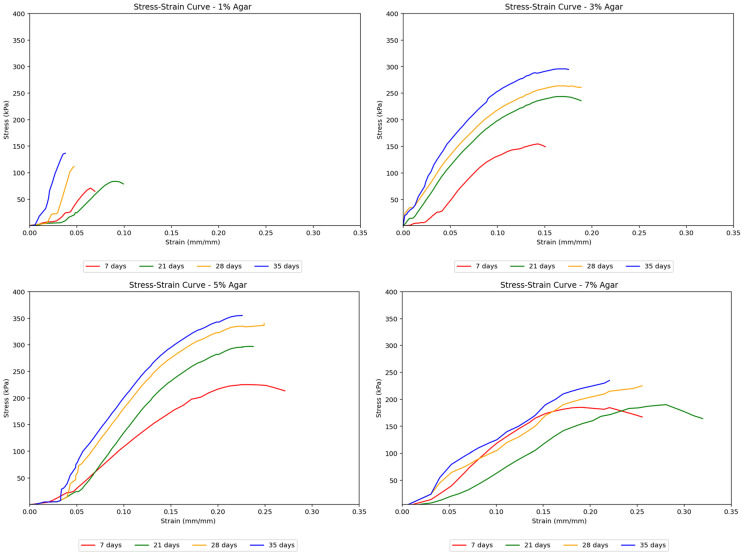
Effect of curing period on the stress–strain curves for agar-treated soil samples.

Agar-treated soils exhibit a smooth transition from elastic to plastic behavior. It reaches the elastic limit and plastic limit relatively early on as compared to that of cement-treated soils. This is due to the highly flexible agar material’s natural plastic, gum-like properties. These samples also showed a more consistent shape in their stress–strain curves across increasing curing times while achieving higher peak strengths with longer curing periods. This suggests that the agar treatment resulted in more ductile soils capable of greater deformation before failure. Figure 6 demonstrates the effect of agar content on stiffness across different curing days.

### 3.3. Effectiveness of Concentration

The study investigated the effectiveness of varying concentrations (1%, 3%, 5%, and 7%) of Portland cement and agar biopolymer on low-plasticity soils’ unconfined compressive strength (UCS). The results indicate that increasing the concentration of cement and agar biopolymer generally led to an increase in the UCS of the treated soil samples compared to the untreated soil (0% concentration).

**Figure 7:**
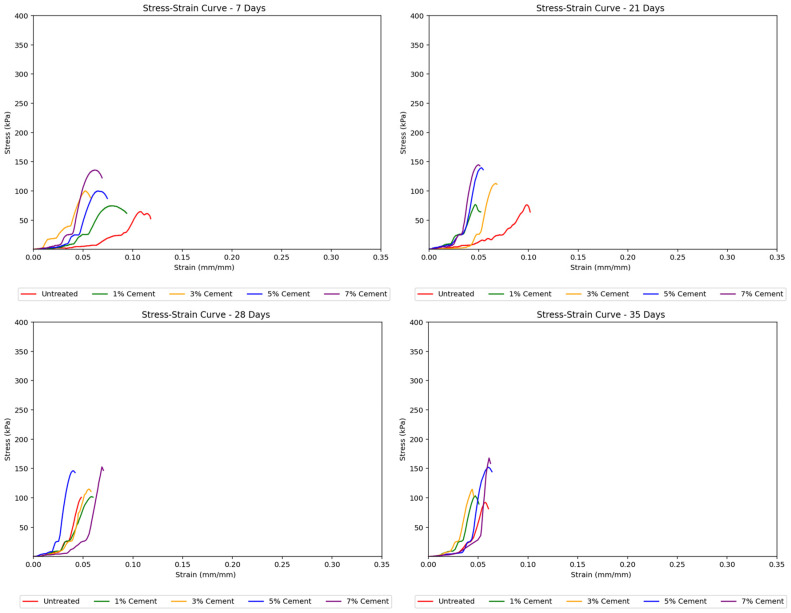
Effect of cement concentration on the stress–strain curves for treated soil samples.

Cement-treated samples generally resulted in higher strengths than untreated soils. The stress–strain curves in Figure 7 for cement-treated soils showed a noticeable transition from elastic to plastic behavior, which occurred later at greater cement concentrations. These curves were also steeper and reached higher peak strengths with increasing cement treatment concentrations, indicating that the soil samples became stronger, albeit more brittle, with more added cement. Sudden jumps in the stress–strain curves suggested abrupt deformation or cracking as the load increased.

**Figure 8:**
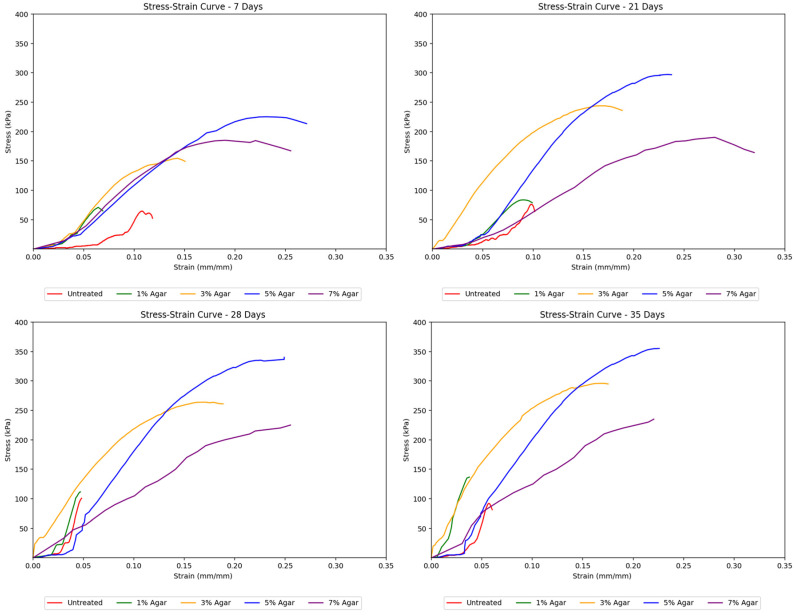
Effect of agar concentration on the stress–strain curves for treated soil samples.

Agar-treated samples also exhibited higher strength than untreated soils. Notably, agar-treated soil samples showed considerably higher strength than cement-treated samples, reaching a peak strength of about 355 kPa at a 5% concentration and 35 days of curing, compared to cement-treated soil samples, which only reached approximately 170 kPa. The stress–strain curves in Figure 8 remained uniform with gentler slopes as the agar concentration increased. This suggests that adding more agar content made the soil behave more ductile, gaining the ability to undergo greater deformation without failure. The uniformity of the curves also implied more gradual deformation in agar-treated samples, unlike the sudden deformation seen in cement-treated samples. In terms of peak strengths, the strength of agar-treated samples consistently increased, reaching its highest point at a 5% concentration, after which the strength began to decrease as the concentration was raised to 7%. This decrease is attributed to the masking effect caused by the agar biopolymers. At lower concentrations, agar biopolymers coat the soil particles and provide cementation between aggregates, which increases the soil’s mechanical strength. However, as the concentration of agar increases, this coating effect starts to “mask” the soil particles, reducing the direct contact and interaction between them. This masking effect is found to reduce the mineral contents of soil, namely montmorillonite and quartz, which are known to increase cohesion between soil particles [28]. Although agar provides cementation, the reduction in the interaction and cohesion between soil particles themselves leads to a point where further addition of agar beyond an optimal concentration (5% in this study) results in a decrease in the unconfined compressive strength (UCS). The agar biopolymers essentially occupy the space around the soil particles, and once all available surfaces are coated, adding more agar content becomes ineffective and even detrimental by hindering particle-to-particle bonding. This leads to the observed peak in strength at 5% concentration, followed by a decrease at 7% concentration.

### 3.4. Morphological and Elemental Analysis of Agar-Treated Soil

**Figure 9:**
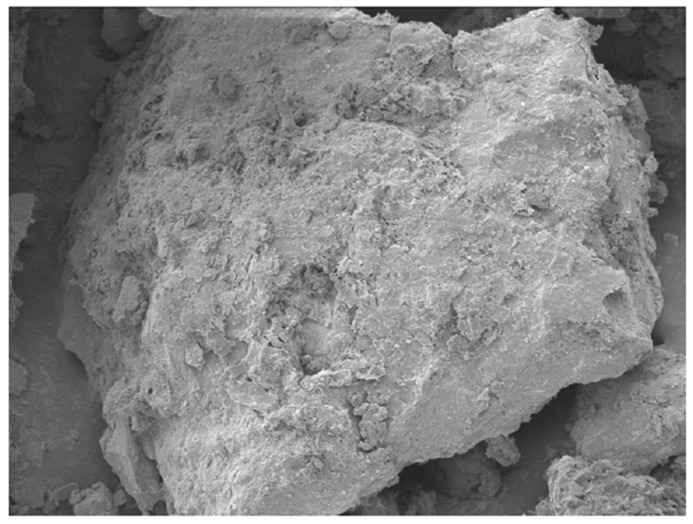
SEM micrographs of soil with 5% agar concentration at 1000*×* magnification.

**Figure 10:**
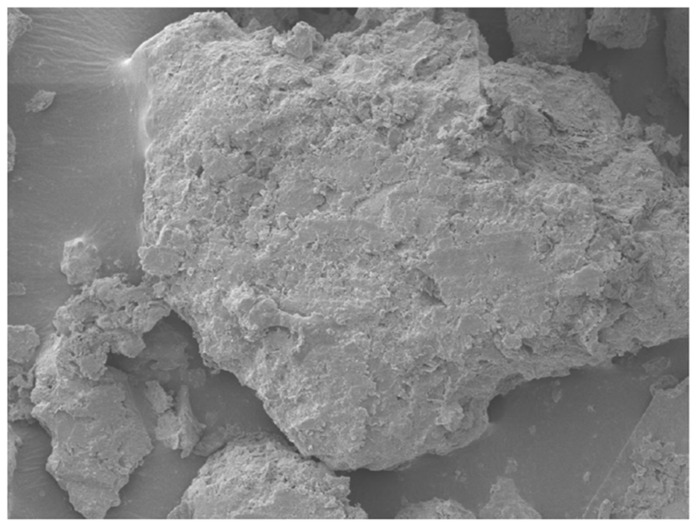
SEM micrographs of soil with 7% agar concentration at 1000*×* magnification.

To understand the mechanisms behind the observed strength behavior of agar-treated soils, a microstructural characterization was performed using Scanning Electron Microscopy (SEM) and Energy Dispersive X-ray Spectroscopy (EDS) on samples treated with 5% and 7% agar concentration. The SEM micrographs in Figure 9 and Figure 10 revealed distinct differences in the surface morphology as a function of agar content.

At 5% agar concentration, the soil particles appeared partially encapsulated by thin biopolymer films, promoting enhanced particle-to-particle bonding while maintaining sufficient mechanical interlocking. Microstructural features such as visible contact between grains and relatively thin biopolymer bridges were observed, conducive to strength improvement.

At 7% agar concentration, a markedly denser and smoother biopolymeric matrix was evident. Soil grains were almost entirely coated, with fewer visible interparticle contacts. The thickening of the biopolymer layer suggests excessive encapsulation, potentially reducing direct mechanical interlocking between soil particles and promoting a softer, more ductile response under loading.

**Figure 11:**
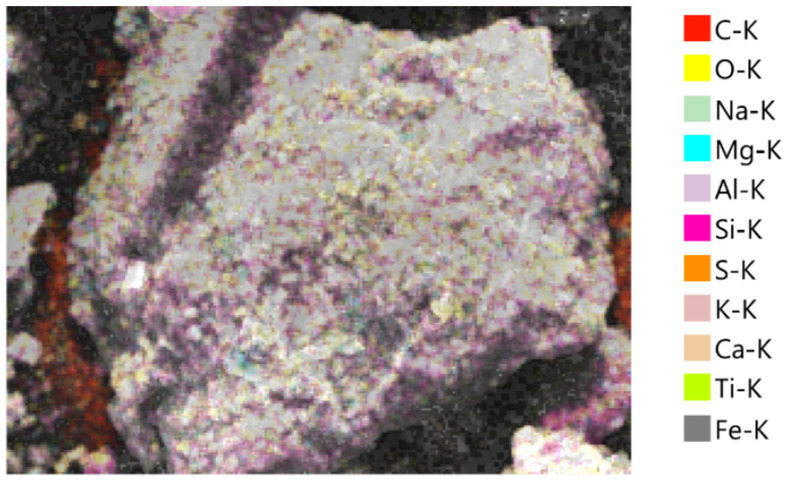
SEM-EDS elemental mapping of soil treated with 5% agar biopolymer-treated soil.

**Figure 12:**
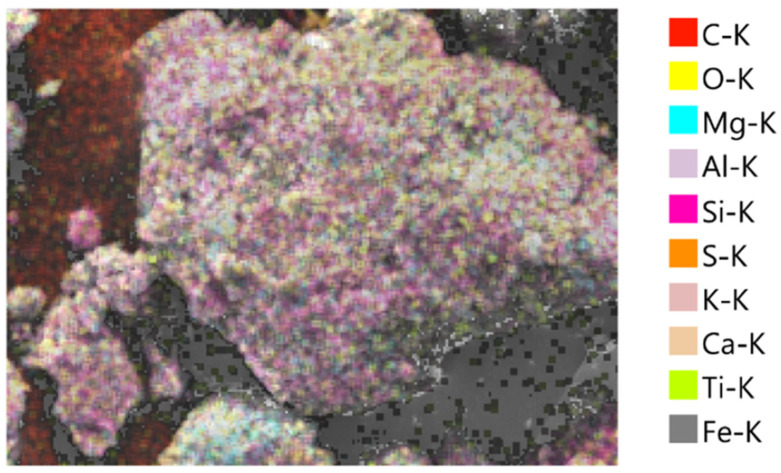
SEM-EDS elemental mapping of soil treated with 7% agar biopolymer-treated soil.

The EDS analyses supported these morphological observations. In the 5% agar-treated sample (Figure 11 and Table 2), the mass percentages were dominated by oxygen (46.51%), carbon (18.88%), and silicon (16.08%), reflecting the mixture of natural soil minerals and the introduced biopolymer. In the 7% agar-treated sample (Figure 12 and Table 3), the carbon content increased significantly to 29.23%, while the oxygen content slightly decreased to 43.74%. The observed increase in carbon content with increasing agar concentration confirms greater biopolymer incorporation into the soil matrix. However, the corresponding morphological evolution from partial coating at 5% to thick encapsulation at 7% offers a microstructural explanation for the mechanical behavior. While moderate biopolymer addition enhances strength by improving cohesion and bridging particles, excessive biopolymer accumulation reduces the effective interparticle friction and mechanical interlocking, leading to a slight decrease in strength beyond the optimum concentration of 5%.

Although this study focused on the morphological characterization of agar-treated soils, a comparison with existing literature on cement-treated soils provides additional context. Hua et al. [40] observed through SEM that calcium–silicate–hydrate (C–S–H) gels formed in cementitious composites exhibit a porous and heterogeneous microstructure, with discrete bonding zones between particles and visible crystalline hydration products. In contrast, the SEM images of agar-treated soils in this study revealed a relatively smoother and denser biopolymer matrix that more uniformly encapsulated soil grains. Unlike the crystalline morphology associated with cement hydration, the agar biopolymer forms a continuous organic film, which improves particle cohesion primarily through adhesion and encapsulation rather than chemical bonding. This distinction highlights the differing stabilization mechanisms between cement and biopolymer treatment, where cement relies on the formation of rigid C–S–H networks while agar stabilization depends on flexible biopolymer encapsulation to enhance soil strength.

### 3.5. Statistical Analysis

**Figure 13:**
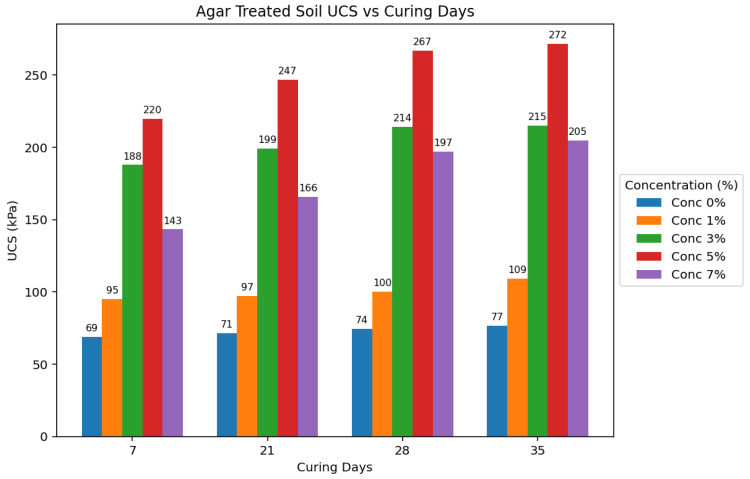
Average effect of curing period on UCS of agar-treated soils per concentration.

**Figure 14:**
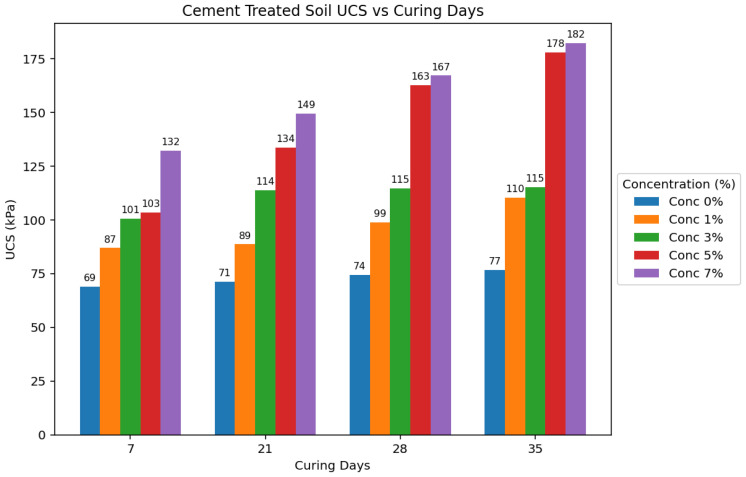
Average effect of curing period on UCS of cement-treated soils per concentration.

This research compared the strength properties of low-plasticity soils treated with agar biopolymers and Portland cement binders to untreated soil samples. The primary method for assessing strength was the Unconfined Compressive Strength (UCS) test, performed after various curing periods (7, 21, 28, and 35 days) and at different concentrations of the binders (1%, 3%, 5%, and 7%). Figure 13 and Figure 14 show the average compressive strengths obtained for cement-treated and agar-treated soils, respectively, for each curing period used in the study.

Agar-treated soils exhibited greater strength than cement-treated soils across all concentrations and curing periods. The peak UCS attained by agar-treated specimens was 271.651 kPa (5% concentration + 35 days of curing). In contrast, the maximum UCS for cement-treated soils was 182.245 kPa (7% concentration + 35 days of curing). Agar-treated soils also yielded significantly stronger samples than cement-treated samples at 3% and 5% concentrations.

**Figure 15:**
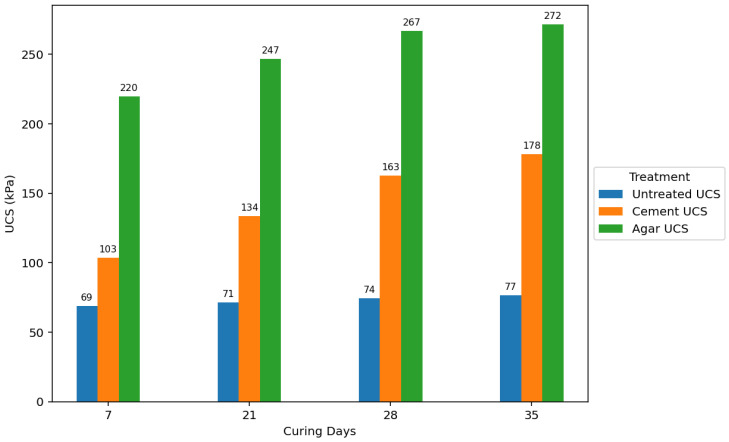
Average effect of curing period on UCS for untreated soils, 5% cement-treated soils, and 5% agar biopolymer-treated soils.

The strength of agar-treated and cement-treated soils also generally increased with longer curing periods. This is attributed to moisture loss over time, leading to increased soil strength. However, agar-treated samples showed a rapid increase in strength during the initial curing stages compared to cement-treated and untreated samples. The study also found that agar-treated samples exhibited higher strength at shorter curing periods than cement-treated samples at longer curing periods. Figure 15 visually compares the average effect of the curing period on UCS for untreated, 5% cement-treated, and 5% agar biopolymer-treated soils, showing a rapid initial strength gain in agar-treated samples. While increased curing time generally improved the strength of all treated soils, agar biopolymer treatment showed a potential for faster initial strength development compared to cement, and the agar concentration was identified as a more influential factor in the final UCS achieved in this study.

**Figure 16:**
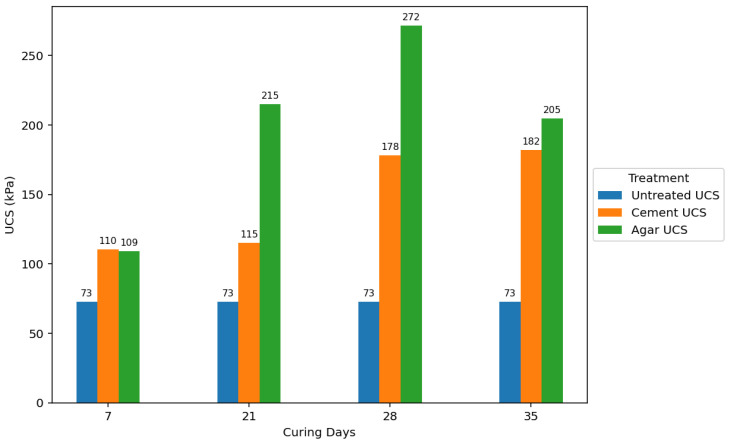
Average effect of concentration on UCS of cement-treated soils, and agar biopolymer-treated soils.

For agar-treated soils, the UCS increased with concentration up to 5%, followed by a decrease in value. This is likely due to the masking effect of the agar biopolymers, hindering effective bonding between all soil particles [28]. The UCS generally increased with increasing cement concentration throughout the tested range for cement-treated soils. Figure 16 further emphasizes that the agar treatment yielded substantially higher UCS values at lower concentrations than cement treatment. This suggests that agar can be more effective in enhancing soil strength with less stabilizing material.

A *t*-test of paired samples was also performed to compare the means of different treatment groups and concentrations and determine if the observed differences in unconfined compressive strength (UCS) were statistically significant. The *t*-test compared the UCS of agar-treated and cement-treated soils at equivalent concentrations (1%, 3%, 5%, and 7%) across various curing periods (7, 21, 28, and 35 days), assuming equal variances. The null hypothesis considers no significant difference in UCS between the agar-treated and cement-treated samples, while the alternative hypothesis indicates a significant difference in UCS. A significance level (alpha, α) of 95% (or 0.05) was used to evaluate the *p*-values obtained from the *t*-tests. The null hypothesis was rejected if the *p*-value was less than α, indicating a statistically significant difference. The results of the *t*-tests are shown in Table 4 and Table 5**.**

Agar-treated soils exhibited higher UCS values than cement-treated soils at the same concentration, especially at 3% and 5%, leading to statistically significant differences. At 1% concentration, the treatments behaved similarly, resulting in insignificant differences. At 7%, the performance of agar remained higher, but the difference was not always significant, possibly due to diminishing returns or material variability at high dosages. Curing time enhanced UCS in both treatments. However, the agar treatment showed superior strength gain at earlier curing stages, likely due to faster physical bonding mechanisms than slower cement hydration.

### 3.6. Formulation of Predictive Models

Regression analysis was performed to test the results and formulate a model that can predict the UCS at any curing day and percentage of agar concentration. A polynomial regression analysis was performed for the UCS results of agar-treated soil to formulate an equation that can predict the UCS at any curing day and percentage agar concentration. Equation 2 represents the predictive model with an R^2^ value of 0.94, indicating that the model fits the data well.

(2) $$UCS=60.945+26.876\left(C\right)+1.504\left(T\right)-4.942{\left(C\right)}^{2}+0.140\left(C\right)\left(T\right)-0.011{\left(T\right)}^{2}$$

where:

*UCS* = unconfined compressive strength (in KPa)

*C* = agar concentration (in %)

*T* = curing time (in days)

A 1% increase in agar concentration increases the UCS by approximately 26.88 kPa, provided a constant curing period. This has a strong positive effect. A 1-day increase in curing time increases UCS by about 1.50 kPa, assuming concentration is constant. This shows a moderate time-based gain. The negative sign in the fourth term (−4.942) implies diminishing returns at higher concentrations. There is an optimal point beyond which the strength gain slows or reverses. The positive interaction between agar concentration and curing time (+0.140) means that increasing both concentration and curing time results in an even greater UCS than when increased independently. The negative quadratic term for curing time (−0.011) indicates a slightly diminishing return on strength gain with longer curing times. The UCS increases with curing time and concentration, but the increase is non-linear. The model can determine optimal treatment parameters for the desired soil strength.

**Figure 17:**
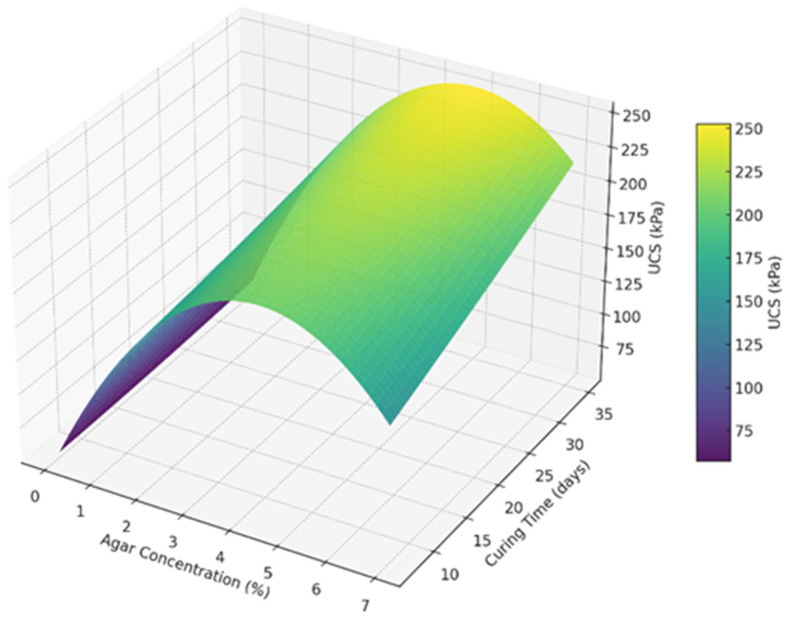
3D surface plot of UCS model against agar concentration and curing time.

A 3-D surface plot shown in Figure 17 was made to visualize the effect of agar concentration and the curing time on the UCS of soil. It is evident from the plot that the UCS increases sharply with agar concentration up to around 3 to 5%. Beyond 5% concentration, the UCS gain tapers off or slightly declines, supporting the diminishing returns observed in the regression model. Longer curing times continue to increase UCS, but with slower growth after around 28 days. The peak strength region lies in the upper-middle range of concentration and time, around 5% agar and 28 to 35 days.

**Figure 18:**
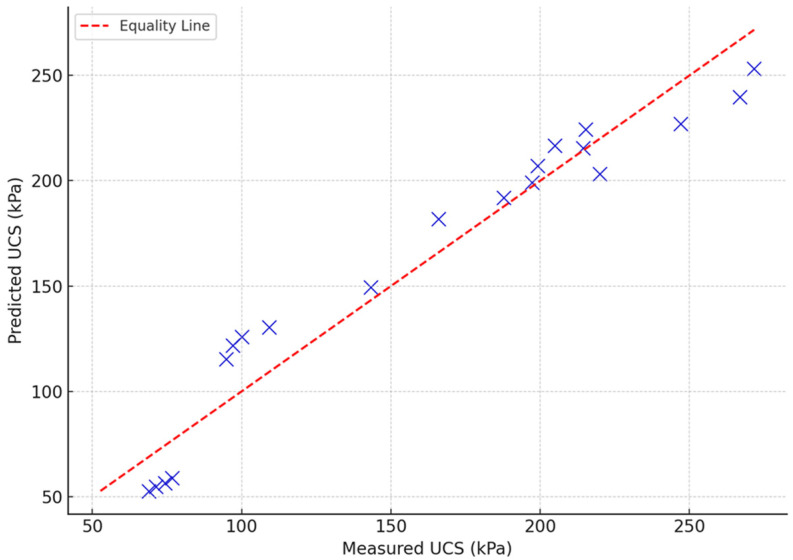
Predicted UCS vs. measured values for the agar-treated soil.

To validate the predictive capabilities of the formulated model, the predicted UCS using Equation (2) was compared with the measured (actual) values and was plotted in a scatter plot as shown in Figure 18. It can be noted that the values are close to the equality line, which indicates that the predicted values can represent the measured values. The proximity of points to the equality line suggests the model’s accuracy. The closer the points are to the line, the better the model fits the data. The model shows strong predictive performance, with most values closely hugging the equality line, reinforcing the previously reported high R^2^ value (~0.94).

## 4. Conclusions

The research compared the effects of agar biopolymer and Portland cement treatments on the unconfined compressive strength (UCS) of these soils across various curing periods and binder concentrations.

Both agar and cement treatments were found to increase the strength of low-plasticity soil compared to untreated soil. This confirms the hypothesis that agar biopolymer has good stabilizing potential. Notably, agar-treated soils generally exhibited higher UCS values than cement-treated soils across the tested conditions. The peak strength achieved with agar was approximately 270 kPa at 5% concentration after 35 days of curing, significantly higher than the peak of around 180 kPa for cement at 7% over the same period. This suggests that, within the tested range, agar can be more effective in enhancing the compressive strength of this type of soil.

This study also identified an optimal agar concentration of 5%, beyond which the compressive strength decreased. This is attributed to a potential masking effect where excess agar might prevent effective bonding between soil particles. On the other hand, cement generally showed increasing strength with concentration.

The SEM-EDS results confirmed that 5% agar concentration provides optimal particle bonding and strength improvement, whereas higher concentrations lead to excessive biopolymer encapsulation, reducing mechanical interlocking and slightly decreasing strength.

Observations of the stress–strain curves and failure modes revealed that agar-treated soils exhibited more ductile behavior, undergoing more deformation before failure, compared to the more brittle cement-treated soils. This could have implications for the ability of the soil to withstand stress without sudden failure.

Regression analysis provided a model that could predict the UCS values of the agar treatment based on concentration and curing time, with line fit plots validating the accuracy of these models within the study parameters. Furthermore, *t*-tests confirmed that the agar content significantly improved UCS compared to untreated soil and often outperformed cement at 3% and 5% concentrations.

This study concludes that agar biopolymers derived from *Gracilaria* seaweed present a promising and potentially more effective, environmentally friendly alternative to Portland cement for stabilizing low-plasticity clay soils, particularly at specific concentrations. This is particularly significant given the environmental concerns associated with cement production. However, the researchers recommend further investigating the long-term effects and performance under various environmental conditions through durability and swelling tests to solidify the feasibility of using agar biopolymers in practical geotechnical applications.

## Figures and Tables

**Table 1:** Physical properties of untreated soil.

	Index Values	ASTM Standard
Specific Gravity	2.64	ASTM D854 [32]
Liquid Limit, %	41	ASTM D4318 [29]
Plastic Limit, %	31	ASTM D4318 [29]
Plasticity Index (PI), %	10	ASTM D4318 [29]
Optimum Moisture Content (OMC), %	19.79	ASTM D698 [33]
Maximum Dry Unit Weight, kN/m^3^	15.72	ASTM D698 [33]
Soil Classification according to USCS	ML (low-plasticity silt)	ASTM D2487 [31]

**Table 2:** Mass and atomic percentage of elemental composition of 5% agar-treated soil.

Element	Line	Mass %	Atom %
C	K	18.88 ± 0.12	27.96 ± 0.18
O	K	46.51 ± 0.20	51.71 ± 0.22
Na	K	2.22 ± 0.04	1.72 ± 0.03
Mg	K	1.38 ± 0.03	1.01 ± 0.02
Al	K	6.73 ± 0.06	4.43 ± 0.04
Si	K	16.08 ± 0.09	10.18 ± 0.06
S	K	0.62 ± 0.02	0.34 ± 0.01
K	K	0.66 ± 0.02	0.30 ± 0.01
Ca	K	0.95 ± 0.03	0.42 ± 0.01
Ti	K	0.35 ± 0.02	0.13 ± 0.01
Fe	K	5.63 ± 0.11	1.79 ± 0.04
Total		100.00	100.00

**Table 3:** Mass and atomic percentage of elemental composition of 7% agar-treated soil.

Element	Line	Mass %	Atom %
C	K	29.23 ± 0.13	40.25 ± 0.19
O	K	43.75 ± 0.21	45.21 ± 0.22
Mg	K	1.91 ± 0.03	1.30 ± 0.02
Al	K	5.23 ± 0.05	3.20 ± 0.03
Si	K	12.54 ± 0.08	7.38 ± 0.05
S	K	0.54 ± 0.02	0.28 ± 0.01
K	K	1.26 ± 0.02	0.53 ± 0.01
Ca	K	1.56 ± 0.04	0.64 ± 0.01
Ti	K	0.47 ± 0.02	0.16 ± 0.01
Fe	K	3.52 ± 0.09	1.04 ± 0.03
Total		100.00	100.00

**Table 4:** Summary of *t*-test results between agar and cement-treated samples.

Curing Days	Concentration (%)	T-Statistic	*p*-Value	Significance
7	1.0	−1.87844	0.13352	Insignificant
	3.0	−21.87639	0.00003	Significant
	5.0	−13.52651	0.00017	Significant
	7.0	−2.99666	0.04007	Significant
21	1.0	−0.94318	0.39899	Insignificant
	3.0	−25.42387	0.00001	Significant
	5.0	−16.76992	0.00007	Significant
	7.0	−1.76669	0.15203	Insignificant
28	1.0	−0.15241	0.88625	Insignificant
	3.0	−18.07330	0.00006	Significant
	5.0	−7.36866	0.00181	Significant
	7.0	−3.32159	0.02933	Significant
35	1.0	0.11204	0.91619	Insignificant
	3.0	−14.26631	0.00014	Significant
	5.0	−6.73660	0.00253	Significant
	7.0	−5.03880	0.00729	Significant

**Table 5:** Summary of *t*-test results between agar-treated and untreated samples.

Curing Days	Concentration (%)	T-Statistic	*p*-Value	Significance
7	1.0	6.0201	0.00384	Significant
	3.0	23.48937	0.00002	Significant
	5.0	17.71475	0.00006	Significant
	7.0	15.93228	0.00009	Significant
21	1.0	3.13128	0.03514	Significant
	3.0	20.2809	0.00003	Significant
	5.0	21.5785	0.00003	Significant
	7.0	10.74017	0.00043	Significant
28	1.0	4.92922	0.00788	Significant
	3.0	23.00029	0.00002	Significant
	5.0	24.59916	0.00002	Significant
	7.0	20.35831	0.00003	Significant
35	1.0	6.08927	0.00368	Significant
	3.0	24.72689	0.00002	Significant
	5.0	13.7655	0.00016	Significant
	7.0	28.52516	0.00001	Significant

## Data Availability

The data presented in this study are available on request from the corresponding author. The data are not publicly available due to privacy.
